# Utility of a Clinical Scoring System (Bandim TB Score and Karnofsky Performance Score) to Assess Mycobacterial Burden in Terms of Cartridge-Based Nucleic Acid Amplification Test (CBNAAT) Cycle Threshold Values Among Pulmonary TB Patients

**DOI:** 10.7759/cureus.50976

**Published:** 2023-12-22

**Authors:** Krishna Sarkar, Bineeta Kashyap, Sharanya LNU, Rajnish K Avasthi, Ashwani Khanna

**Affiliations:** 1 Department of Microbiology, University College of Medical Sciences, Delhi, IND; 2 Medicine, Lady Hardinge Medical College, Delhi, IND; 3 Department of Medicine, University College of Medical Sciences, Delhi, IND; 4 Pulmonary Medicine, Lok Nayak Hospital, Delhi, IND

**Keywords:** cartridge-based nucleic acid amplification test (cbnaat), infectious disease diagnosis, clinical features, genexpert mycobacterium tuberculosis/rifampin (mtb/rif) assay, active pulmonary tuberculosis

## Abstract

Aim: Tuberculosis (TB) continues to be a global public health problem. Physicians fail to clearly interpret cycle threshold (Ct) values as a measure of mycobacterial burden due to the paucity of literature correlating Ct values with the clinical scoring. This study aims to correlate the clinical scoring parameters (Bandim TB score and Karnofsky Performance score (KPS)) with Ct values obtained by Cartridge-Based Nucleic Acid Amplification Test (CBNAAT).

Materials and methods: The study spanned from November 2019 to October 2021, during which a total of 40 cases were recruited. These cases were identified as pulmonary TB patients based on Ziehl-Neelsen staining for acid-fast bacilli and/or the GeneXpert MTB/RIF assay. Bandim TB scores and KPSs were recorded using standardized questionnaires.

Results: There was a strong negative correlation between Bandim TB score and Ct value (mean), and this correlation was statistically significant (rho = -0.82, p < 0.001). There was a moderate positive correlation between KPS and Ct value (mean), and this correlation was statistically significant (rho = 0.57, p < 0.001).

Conclusion: No literature has compared Bandim TB score and KPS with the Ct values obtained by CBNAAT for pulmonary TB. Thus, the knowledge on the proper utilization of CBNAAT cycle threshold values and its correlation with clinical scoring parameters will help clinicians in the early identification and prompt initiation of appropriate treatment.

## Introduction

Tuberculosis (TB) continues to be a global public health problem despite significant progress, and it is the leading cause of deaths from a single infectious agent (ranking above HIV/AIDS). Approximately 7.1 million people with TB were reported to have been newly diagnosed and notified in 2019 globally [1]. For early detection of mycobacterial growth, liquid culture techniques were developed, but the turnaround time of 21 days is still quite long for a diagnostic test to control transmission [2]. A wide array of molecular tests is available for the detection of *Mycobacterium tuberculosis*. Some tests are available as point-of-care tests in peripheral healthcare settings. The WHO-endorsed nucleic acid amplification techniques include the Xpert MTB/RIF assay, line probe assays (LPAs), Xpert Ultra, loop-mediated isothermal amplification (LAMP), and Truenat [3]. The WHO endorsed nucleic acid amplification tests for the first time in 2008 for use in resource-limited settings to rise to the challenge of global drug resistance epidemic. The Xpert MTB/RIF assay has been a boon for TB control, as it can provide results within two hours. It can detect as low as 100 colony-forming unit (CFU) per sample, improving the sensitivity for patients with low bacillary burden compared to smear microscopy [4]. It provides a cycle threshold (Ct) value, which is the number of PCR cycles required to detect *M. tuberculosis*, where each subsequent cycle represents 50% less starting material than the last. It thereby provides a semi-quantitative result of the bacillary burden, where higher Ct values reflect a lower bacillary burden [5]. A semi-quantitative estimate of the concentration of bacilli can be defined by the Ct range (>28 = very low, 22-28 = low, 16-22 = medium, <16 = high) [6]. It utilizes real-time PCR technology to diagnose TB and detects rifampicin resistance using unprocessed clinical specimens.

Based on the available resources in the hospital settings, clinical scores are needed to evaluate the patient outcome response. Several scoring systems are available for diagnostic purposes, but a simple clinical scoring system for repeated clinical evaluation is lacking. Scoring symptoms, signs, and anthropometric values of patients would be helpful to describe the prognosis of TB treatment.

Based on readily available clinical data and diagnostic investigations, the Bandim Tuberculosis Score and Karnofsky Performance Score (KPS) have been developed in resource-constrained settings [7]. The Bandim TB score encompasses five self-reported symptoms and six signs. Each clinical variable contributes one point, with an additional point counted if the body mass index falls below 16 kg/m^2^ or if the mid-upper arm circumference (MUAC) is less than 200 mm. The maximum score is 13. Patients are categorized into three severity classes: SC I (score 0-5), SC II (score 6-7), and SC III (score 8-13 points).

On the other hand, KPS is a subjective tool that assesses a patient's performance on a scale from 0% to 100% based on their capacity to carry out daily activities, work, their need for assistance, and the presence of disease-related symptoms. Patients are assigned to three severity classes: SC I (80-100%), SC II (50-70%), and SC III (0-40%), as determined by their performance percentage [7].

This study aims to correlate the clinical scoring parameters, namely, Bandim TB score and KPS with Ct values obtained by CBNAAT, thus providing an excellent tool for clinicians in rapid diagnosis and appropriate management of TB.

## Materials and methods

Ethical consideration

This study received approval from the Institutional Review Board of the Institutional Ethics Committee-Human Research at the University College of Medical Sciences (IEC-HR/2019/41/68) and adhered to the principles outlined in the Declaration of Helsinki. Written informed consent was obtained from all participants.

Study design

We conducted a cross-sectional study involving the Departments of Microbiology and Medicine and the DOTS Centre. The study spanned from November 2019 to October 2021, during which we recruited a total of 40 cases. These cases were identified as pulmonary TB patients based on Ziehl-Neelsen staining for acid-fast bacilli and/or the GeneXpert MTB/RIF assay (Cepheid Inc., Sunnyvale, California).

Data collection

We utilized standardized questionnaires to record the Bandim TB score (Table 1) and the KPS (Table 2) of the participants [8,9]. In addition, we documented signs and symptoms following comprehensive history-taking and clinical examinations.

**Table 1:** Bandim TB score BMI: body mass index, MUAC: mid-upper arm circumference

Parameters	Points assigned
Cough	1
Hemoptysis	1
Dyspnea	1
Chest pain	1
Night sweating	1
Anaemic conjunctiva	1
Tachycardia	1
Positive finding at lung auscultation	1
Axillary temperature > 37℃	1
BMI*< 18	1
BMI*< 16	1
MUAC**<220	1
MUAC**<200	1

**Table 2:** Karnofsky performance status scale

Condition	Performance status %	Comments
Capable of engaging in regular daily activities and employment without the need for specialized assistance	100	Indicates normal function with no complaints or evidence of disease
	90	Reflects the ability to engage in normal activities with minor signs or symptoms of disease
	80	Suggests that normal activity is possible but with effort and the presence of some disease-related signs or symptoms
Not capable of working but can manage to reside at home and attend to most personal needs, although some level of assistance is required to varying degrees	70	Denotes the ability to care for oneself but an inability to perform normal activities or work
	60	Signifies the need for occasional assistance while still maintaining personal care abilities
	50	Indicates a significant need for assistance and frequent medical care
Unable to self-care, necessitating an equivalent level of care typically provided in institutional or hospital settings, especially when the disease is advancing rapidly	40	Implies a state of disability, necessitating special care and assistance
	30	Represents severe disability, with a need for hospitalization even if death is not imminent
	20	Indicates that hospitalization is necessary, and active supportive treatment is crucial due to a very sick condition
	10	Characterizes a moribund state with rapidly progressing fatal processes
	0	Signifies death

Data analysis

We conducted data analysis using IBM SPSS Statistics for Windows, version 23 (released 2015; IBM Corp., Armonk, New York, United States). To assess the correlation between Ct values and clinical scores, we calculated the Spearman correlation coefficient. For non-normally distributed data, we employed the Kruskal-Wallis test, which is a suitable non-parametric test. We considered statistical significance when the p-value was less than 0.05.

## Results

Sputum samples from a total of 40 patients were subjected to CBNAAT. Clinical scores recorded using the questionnaires were classified according to severity. Twenty-five (62.5%) of the total patients were males and 15 (37.5%) were females. The male-to-female ratio was 1.6:1. The male to female ratio was 1.6:1.

Correlation of Bandim TB score with Ct value (mean)

**Figure 1:**
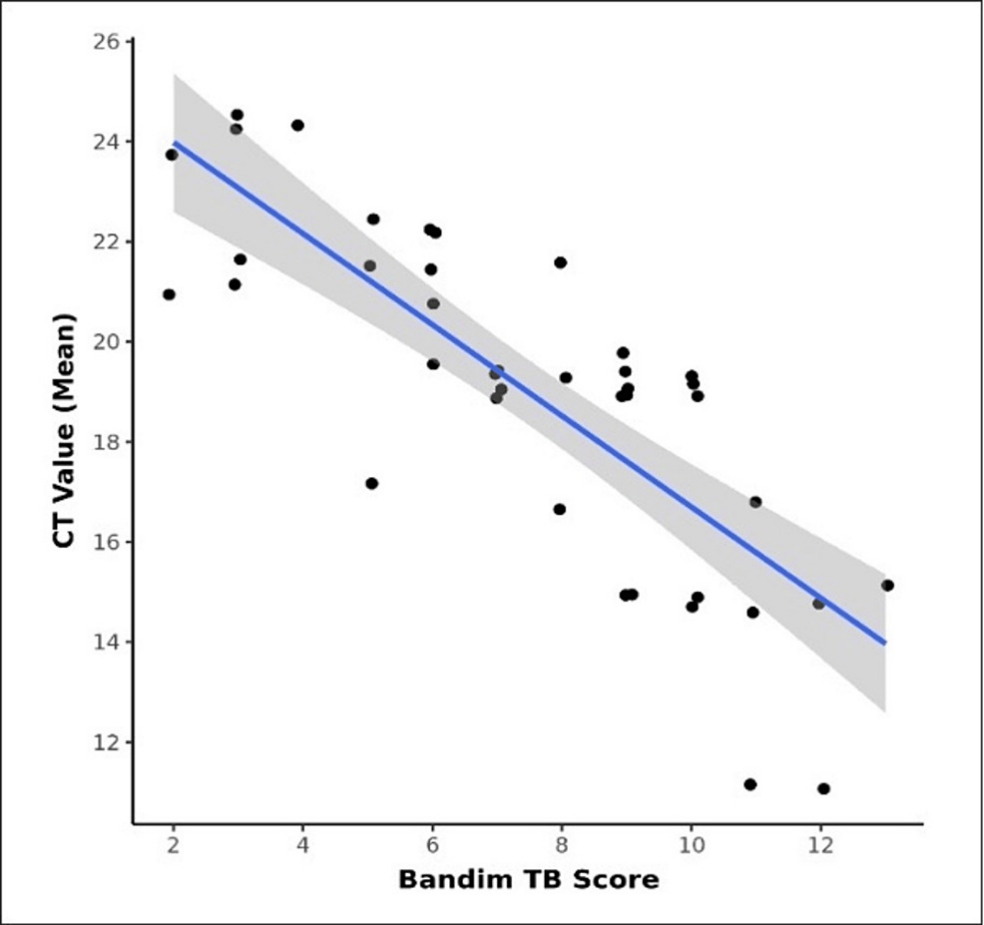
Correlation between the Bandim TB score and Ct value (mean) (n = 40)

We observed a robust negative correlation between the Bandim TB score and Ct value (mean), as illustrated in Figure 1. This correlation was statistically significant (rho = -0.82, p < 0.001). Specifically, for each incremental increase of one unit in the Bandim TB score, the Ct value (mean) decreased by 0.91 units. Table 3 depicts the comparison of three subgroups of Bandim score severity in terms of the Ct value (mean).

**Table 3:** Comparison of the three subgroups of Bandim score severity in terms of Ct value (mean) (n = 40) Ct: cycle threshold, SD: standard deviation, IQR: interquartile range

CT value (mean)	Bandim score severity			Kruskal-Wallis test	
	0 to 5	6 to 7	8 to 13	χ2	p-value
Mean (SD)	22.17 (2.26)	20.34 (1.35)	16.86 (2.88)	20.820	<0.001
Median (IQR)	22.01 (21.22-24.11)	19.52 (19.26-21.48)	16.74 (14.9-19.08)		
Range	17.12 - 24.56	18.94 - 22.32	11.08 - 21.58		

Correlation of the KPS and Ct value (mean)

**Figure 2:**
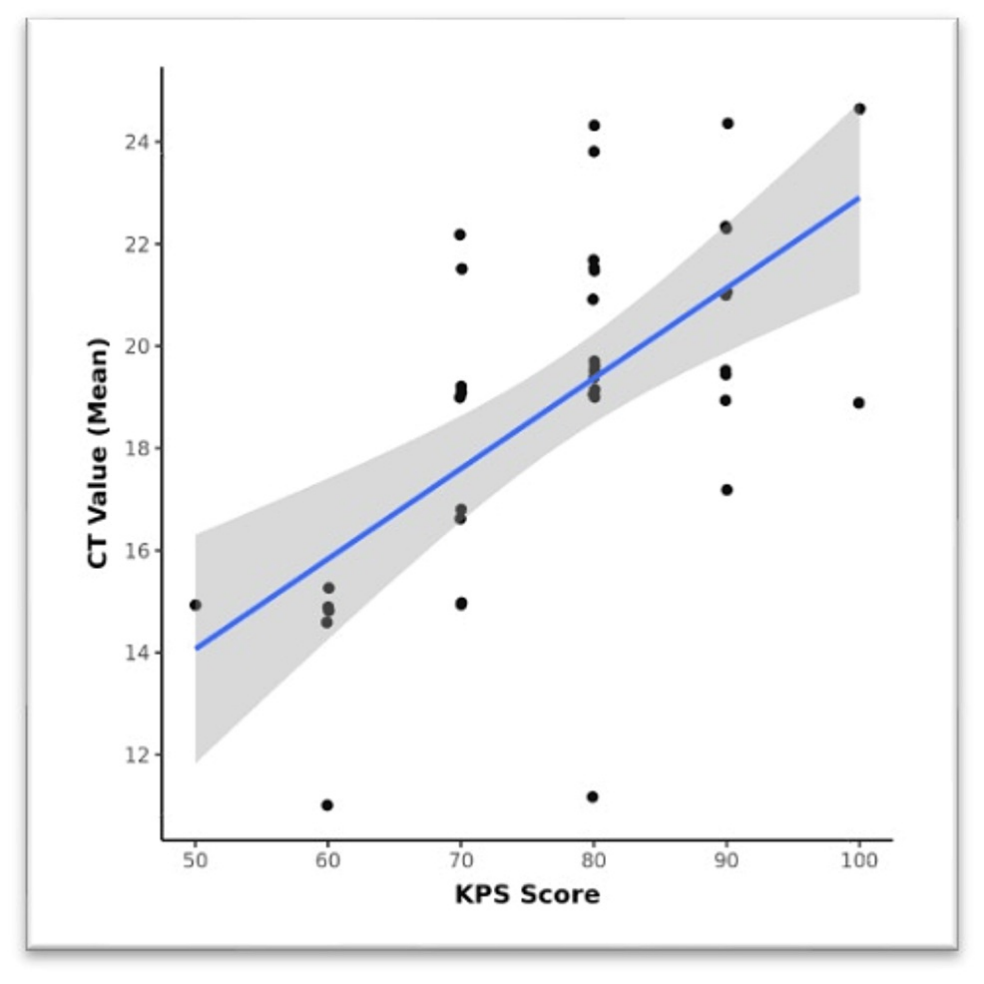
Correlation between the KPS and Ct value (mean) (n = 40)

Figure 2 depicts the correlation of the KPS and Ct value (mean). There was a moderate positive correlation between the KPS and Ct value (mean), and this correlation was statistically significant (rho = 0.57, p < 0.001). For every one unit increase in the KPS, the Ct value (mean) increases by 0.18 units. 

**Figure 3:**
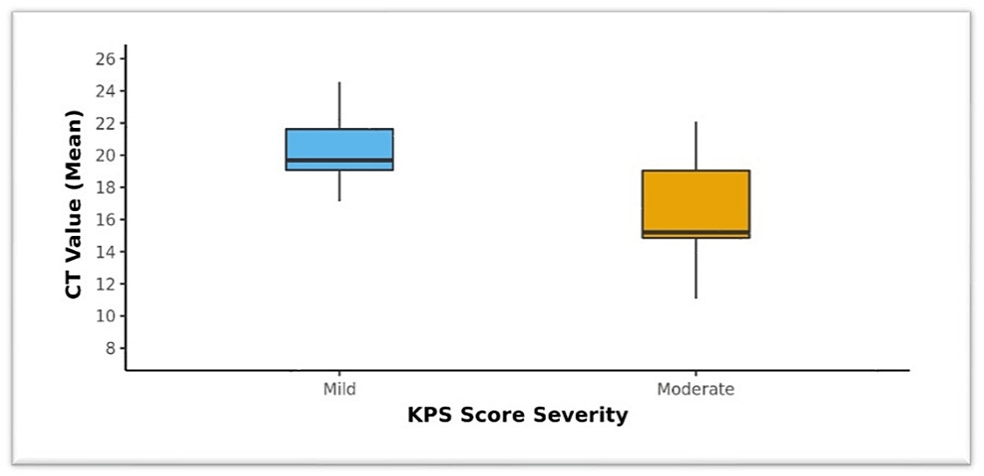
Comparison of the KPS severity in terms of the Ct value (mean) (n = 40)

Figure 3 shows the box-and-whisker plot depicting the comparison of sub-groups of KPS severity in terms of the Ct value (mean). The middle horizontal line represents the median Ct value (mean); the upper and lower bounds of the box represent the 75th and 25th centiles of the Ct value (mean), respectively; and the upper and lower extents of the whiskers represent the Tukey limits for the Ct value (Mean) in each of the groups. 

## Discussion

TB continues to spread globally, and there is a paucity of literature on the clinical scoring systems among pulmonary TB cases. In our study, the Ct value (mean) was significantly associated (p < 0.05) with the Bandim TB score and KPS. Our study documented a statistically significant and strong negative correlation (Spearman correlation coefficient = -0.8) between the Bandim TB score and Ct value (mean). The strong negative correlation between the Bandim TB score and Ct value suggests that the Bandim score can serve as a reliable indicator of TB severity, aligning with the bacterial load in patients. This finding supports the clinical utility of the Bandim score for assessing disease severity and monitoring treatment response. The comparison of Ct values among the three Bandim score severity subgroups further strengthens the association between the Bandim score and disease severity. The significant difference observed in Ct values indicates that the severity subgroups exhibit distinct levels of disease progression and bacterial burden. The decreasing trend in median Ct values across the severity subgroups indicates a progressive increase in disease severity, corroborating the validity of the Bandim score in capturing the disease progression accurately.

Wejse et al. in their study on pulmonary TB assessed the outcome of treatment for smear-negative patients using the Bandim TB score. He also established that the score had a good predictive capacity and was sensitive to changes during the treatment of pulmonary TB. The same study documented a mortality of 21% for patients with a TB score of >8 during the eight months' treatment as compared to 11% for the TB score <8 [8]. In our study, however, we could not follow up the cases to correlate the clinical scoring with the mortality or the outcome of the disease, due to the fact that our hospital was a dedicated COVID center during the pandemic, which led to the disruption of the routine patient care activities. Janols et al. in their study evaluated a clinical scoring system (TB score) during the intensive phase of treatment in pulmonary TB patients and established a significant decline in the TB score results at two weeks after starting TB treatment [9]. Aunsborg et al. in their study on pulmonary TB among HIV-infected patients concluded that systematic screening using the TB score resulted in a three-fold increase in the detection of TB coinfection among HIV-infected patients [10]. In a study conducted in Indonesia, the modified Bandim scoring system was used to assess the severity class of MDR-TB patients and established the association with specific sequences of the full ESX A gene from MDR-TB sputum isolates [11].

Limited studies were done on the KPS. This scoring system was initially used to evaluate the treatment response among the patients receiving chemotherapeutic agents and overall assess the quality of life. Our study documented a moderate positive correlation (Spearman's correlation coefficient = 0.6) between the KPS and Ct value (mean), and this correlation was statistically significant (rho = 0.57, p < 0.001). This result suggests that there is a relationship between the functional status of the patients, as indicated by the KPS, and the quantitative measure of disease severity, represented by the Ct value (mean). The positive correlation observed implies that as the KPS score increases, indicating a better functional status, the Ct value (mean) also tends to increase. In other words, patients with higher KPS scores tend to have higher Ct values, indicating a lower bacterial load and less severe disease. This finding is consistent with previous research indicating that patients with a better functional status often exhibit milder disease manifestations and lower bacterial burdens. The magnitude of the correlation coefficient between the KPS and Ct value (mean) indicates that while there is a discernible relationship, other factors may also influence the Ct value (mean) besides the functional status of the patients. These factors may include variations in immune response, host genetics, treatment adherence, and comorbidities. Further investigations exploring these factors would provide a more comprehensive understanding of the determinants of disease severity in TB. The statistically significant p-value (p < 0.001) indicates that the observed correlation between the KPS and Ct value (mean) is unlikely to have occurred by chance. Therefore, this correlation can be considered robust and meaningful in the context of assessing disease severity in TB patients. The box-and-whisker plot demonstrates a clear trend in Ct values (mean) across the KPS severity subgroups. This suggests that patients with a lower functional status, reflected in lower KPS scores, tend to have higher bacterial burdens and more severe disease, as indicated by lower Ct values (mean). The box-and-whisker plot provides additional insights into the distribution and variability of Ct values (mean) across KPS severity subgroups, highlighting the importance of considering multiple factors when assessing disease severity in TB patients. Rudolf et al. in their study among pulmonary TB patients in Bissau evaluated the reliability of the Bandim TB score and compared with the KPS [12].

On a global scale, TB remains a significant public health challenge, with a substantial disease burden. The challenge of early diagnosis persists and poses a major obstacle to effective disease management. To our current knowledge, there is a lack of literature that directly compares the Bandim TB score and KPS score with Ct values obtained through CBNAAT for pulmonary TB. Consequently, understanding the appropriate utilization of CBNAAT cycle threshold values and their association with clinical scoring parameters can greatly assist healthcare professionals in identifying cases early and promptly initiating the appropriate treatment.

## Conclusions

Our study demonstrates the significance of the Bandim TB score and KPS in assessing disease severity in TB patients. The correlations with Ct values provide valuable insights into disease progression and bacterial burden. Understanding the correlation between clinical scoring parameters and Ct values can aid clinicians in the early identification and prompt initiation of appropriate treatment for TB patients. Further research is warranted to validate these findings in larger cohorts and investigate the impact of clinical scoring systems on treatment outcomes and mortality in TB.
